# SAMLDroid: A Static Taint Analysis and Machine Learning Combined High-Accuracy Method for Identifying Android Apps with Location Privacy Leakage Risks

**DOI:** 10.3390/e23111489

**Published:** 2021-11-10

**Authors:** Guangwu Hu, Bin Zhang, Xi Xiao, Weizhe Zhang, Long Liao, Ying Zhou, Xia Yan

**Affiliations:** 1 School of Computers, Shenzhen Institute of Information Technology, Shenzhen 518172, China; hugw@sziit.edu.cn (G.H.); liaolong@sziit.edu.cn (L.L.); yanxia@sziit.edu.cn (X.Y.); 2 Peng Cheng National Laboratory, Department of New Networks, Shenzhen 518000, China; xiaox@sz.tsinghua.edu.cn (X.X.); wzzhang@hit.edu.cn (W.Z.); zhou.ying@pcl.ac.cn (Y.Z.); 3 Information Technology Division, Tsinghua Shenzhen International Graduate School, Shenzhen 518055, China; 4 School of Computer Science and Technology, Harbin Institute of Technology, Harbin 150001, China

**Keywords:** android, machine learning, static taint analysis, dynamic taint analysis, location privacy protection

## Abstract

Insecure applications (apps) are increasingly used to steal users’ location information for illegal purposes, which has aroused great concern in recent years. Although the existing methods, i.e., static and dynamic taint analysis, have shown great merit for identifying such apps, which mainly rely on statically analyzing source code or dynamically monitoring the location data flow, identification accuracy is still under research, since the analysis results contain a certain false positive or true negative rate. In order to improve the accuracy and reduce the misjudging rate in the process of vetting suspicious apps, this paper proposes SAMLDroid, a combined method of static code analysis and machine learning for identifying Android apps with location privacy leakage, which can effectively improve the identification rate compared with existing methods. SAMLDroid first uses static analysis to scrutinize source code to investigate apps with location acquiring intentions. Then it exploits a well-trained classifier and integrates an app’s multiple features to dynamically analyze the pattern and deliver the final verdict about the app’s property. Finally, it is proved by conducting experiments, that the accuracy rate of SAMLDroid is up to 98.4%, which is nearly 20% higher than Apparecium.

## 1. Introduction

Recently, with the rise of the mobile internet, location sharing has become an essential and important service, i.e., locating, location sharing, navigation, and finding nearby points of interest (PoIs). According to the survey this study conducted on 20 mobile social applications (apps), e.g., WeChat, Twitter, WhatsApp, Sina Weibo, all involve users’ location sharing or retrieving operations. Although location-based services (LBS) provide great convenience to mobile social network users, location privacy leakage has also aroused great concern because of its potential risks. With users’ geographic location data, perpetrators can not only make precision marketing, but also carry out a variety of attacks, such as long-term statistical attacks, regional statistical attacks, and even personal safety threats.

From the aspect of terminals, the location privacy leakage mainly refers to mobile apps containing backdoors or rogue designs that can be used to obtain a user’s location information without their permission, and bypass the operational system’s security check, so that these apps can send out privacy data via different means, for example, log files, short message service (SMS), and socket communication. In fact, currently a large portion of mobile apps request location access permission from users upon installation. Once permission is granted, these apps arbitrarily utilize users’ location data without their permission. For users, it is hard to know how these apps will deal with their location data, the purposes it will be used for, and who has the privilege to access it.

Existing methods for detecting such malicious apps or malwares with location leakage risks can be divided into two types: static taint analysis and dynamic behavior analysis. Static taint analysis decompiles an app’s source code and analyzes features from the code, e.g., function call sequences and location information flows, which can facilitate detection methods to determine whether the app has a location leakage risk, or not. Dynamic behavior analysis simulates users’ behavior and monitors app reactions in runtime, and can be used to analyze the location data flow from location-retrieving APIs. Although these two methods both have merit, they share a common defect in that they contain a portion of misjudging rate, since some malwares stealthily utilize users’ location data to imitate patterns of behavior similar to normal apps. Moreover, some malwares employ elusion techniques, e.g., dynamic code loading, encryption and native code exploit, to avoid detection. Therefore, it is challenging to distinguish malwares from normal apps, with a high recognition rate.

In order to improve the identification accuracy for malwares with location privacy leakage, this paper proposes SAMLDroid, a combined method of static taint analysis and machine learning for Android platforms. As opposed to existing methods, static code analysis is adopted not only for the merit of fast speed, but also to bring machine learning techniques into the dynamic analysis phase in order to lower the misjudging rate. Briefly, SAMLDroid first uses static taint analysis to scrutinize the target app’s source code and check for the existence of a data flow from the location data, by retrieving APIs from those sent out of the terminals. If it detects this, the app will be transferred to the second phase for dynamic behavior inspection, and the classifier will output the final verdict. Random forest (RF) algorithm was chosen as the classifier, from six candidate machine learning algorithms, and trained with a large number of app samples. Finally, the well-trained classifier outputs a binary result (0 or 1) which indicates that the target app is benign or malicious. The great merit in this method is that it dramatically decreases the misjudging rate compared with existing static or dynamic analysis methods.

The main contributions of this paper are as follows:

(1) The SAMLDroid method, that combines static taint analysis and machine learning to examine whether Android apps have location leakage risks or not, improves upon the deficiency of either static or dynamic analysis with regard to false positive or true negative misjudging rates;
(2) The design of a high-accuracy classifier model that takes apps’ multi-dimensional features into account and adopts the Bayesian network which includes six machine learning algorithms as well as six indicators, to evaluate their performance and choose the best indicator for final evaluation. To the best of the authors’ knowledge, SAMLDroid is the first attempt to use diverse features to train the classifier and evaluate the best indicator for the final verdict;
(3) A series of experiments were conducted on a large dataset containing 24,997 app samples. They resulted in showing that the accuracy of SAMLDroid reaches up to 98.4%, which is nearly 20% higher than Apparecium.

This remaining paper is organized as follows: In Section 2, related work is summarized to offer readers a quick understanding about this subject. In Section 3, the system architecture and its components are elaborated. The method is evaluated in Section 4. Finally, Section 5 concludes the paper and suggests future lines of research.

## 2. Related Work

In view of the great hazard of location leakage risks with insecure apps, many meritorious solutions have been proposed, which mainly focus on three aspects: mobile client side, server side and the communication protocol design between them. On the server side, researchers have solved this problem mainly from the aspect of concealing/confusing location data (e.g., mixed area [1,2]) and mixing the relationship between users and their locations (e.g., k-anonymous [3,4]), while the protocol design has addressed this problem through concepts of encrypting sensitive location data [5] or protecting location query protocol [6]. However, on the client side, researchers have mainly focused on how to distinguish malicious apps from benign apps since they all need to invoke location-related APIs to achieve their purposes, which can be categorized into two types: static analysis and dynamic analysis for Android package (APK) files.

### 2.1. Static Analysis

Static analysis or static taint analysis generally decompiles APK files and analyzes the behavior features based on the decompiled source code. The highly focused features, such as function call sequences and information flows, help to determine the apps’ features. Specifically, this method or related tool usually first defines the source (where APIs access location data) and sink points (where APIs send location data out of the system). Then it analyzes the source code and examines whether there is a sensitive data flow from the source to the sink points. Relying on this result, users can label the target apps as benign or malicious. Much effort has been made, with meritorious results, based on this idea.

Androguard [7] is a basic static taint analysis tool from Google, which can be used to analyze the control flows in APK files and determine whether there is a data stream from the source to the sink points. Similarly, by tracking privacy-getting and privacy-sending actions, Flowdroid [8] traces target apps’ status in every key step and models all transitive relationships in the entire lifecycle, being the first comprehensive static analysis tool based on context, location data flows and app’s lifecycle. Meanwhile, to achieve a highly accurate analysis result, Brox [9] presents an inter-procedural data flow analysis framework which integrates flow sensitive, context-sensitive and inter-procedural techniques together, to perform taint analysis. In addition, Brox comprehensively defines a classified algorithm to evaluate potential privacy-leaking actions, which achieves the purpose of classification speed-up.

To evade the detection means mentioned above, some apps exploit Android inter-component communication (ICC) to illegally retrieve location privacy, because the sensitive data exchanged across components are hardly able to be detected. In order to check such rogue apps, Amandroid [10] and IccTA [11] differentiate vicious privacy leakage actions among the legal information exchange by tracking both control and data flows across different apps’ components and designing a comprehensive assessment algorithm with various benchmarks. Compared with normal static taint analysis methods, more covert leakage actions can be detected. For example, IccTA’s experiment showed the detection of 2395 ICC leaks in 337 apps in a set of 15,000 Google Play apps stores. Furthermore, Iccchecker [12] leveraged the context information triggered by sensitive behaviors and set up a classification algorithm with multiple benchmarks, identifying four apps with ICC-based privacy leakage actions among 168 Google Play apps (2.3%) and 31 malwares from 49 samples (63.3%).

In addition, StubDroid [13] proposed the first automated classification method for precisely and efficiently inferring malwares by automatically constructing app summaries. DroidSafe [14] designed a comprehensive, accurate, and precise classification model for Android apps detection. Lastly, Apparecium [15] provided the authors of this study with intense inspiration in terms of static stain analysis, by revealing all possible data flows from arbitrary data sources to sinks.

### 2.2. Dynamic Analysis

As opposed to static analysis that only examines apps’ source code to find privacy leakage clues, dynamic analysis simulates users’ behavior and monitors apps’ reactions to reveal data flows from all sources and evaluate privacy leakage under apps’ running situations. Compared with static analysis, dynamic analysis can achieve more precise results since it can simulate many real scenarios. However, the disadvantage is a much higher cost in computing and resource consumption.

As the classic representative, TaintDroid [16] achieves this purpose by monitoring app status in a virtual Android running environment. Further, as the extension version of TaintDroid, QuantDroid [17] achieves the same purpose by monitoring the information leakage rate and stopping the app when it reaches a designated threshold. Additionally, it conducts dynamic taint tagging and builds an information flow graph to dynamically examine flows between sources and sinks. Meanwhile, MADAM [18] detects and stops malicious behaviors by monitoring behaviors in both apps and users, and creates features at four levels: kernel, application, user and package.

### 2.3. Hybrid Analysis

In the static and dynamic behavior hybrid detection methods, Monet [19] combined “runtime” behaviors with “static structures” to exploit malware properties, and showed that the runtime behaviors of a malware’s scoring functions were similar within a malware family. As a result, a behavior pattern in a malware family can be summarized. Lastly, the DeepFlow [20] proposal used FlowDroid as its basis and extracted all data flows from arbitrary sensitive sources to sinks. At the same time, it also applied dynamic Bayesian network (DBN) to analyze the high-level features, which achieved good classification results.

In summary, static analysis is fast and efficient because it only needs to analyze apps’ source code. Even though dynamic analysis is much more precise, it is time and resource consuming since it relies on simulator or analog equipment to simulate user behavior and monitor apps’ reactions in runtime. Nevertheless, both share a common defect in that they contain a relatively high misjudging rate.

## 3. System Descriptions

In order to improve the recognition rate for identifying malwares with location privacy leakage risks and consider the pros and cons of the static and dynamic taint analysis methods, a novel method, SAMLDroid, is proposed. It combines static and dynamic analysis and involves machine learning techniques in the dynamic analysis phase to meet high-accuracy recognition rate requirements.

### 3.1. System Overview

Although location privacy leakage brings huge risks to users, users can hardly perceive the rogue behaviors of stealthy GPS data. This is because they have similar patterns to normal apps that provide LBS, such as news apps that push local news to certain people within an area, and music apps that push users to a nearby user list that may have the same interests. Therefore, the challenge here is how to distinguish malwares from benign apps in terms of behavior patterns. The main difference between the patterns is the way in which location data are acquired and sent out, and even though the existing methods of static source code analysis and dynamic behavior analysis have made contributions, there is still a large scope for improvement.

**Figure 1:**
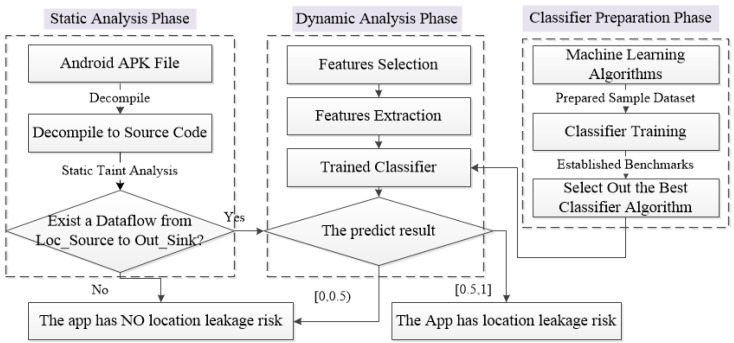
System process diagram.

In consideration of the pros and cons of the static and dynamic taint methods, the proposed system joins these methods, with a division of the whole process into two phases. Briefly, the first static analysis phase takes charge of analyzing target apps from a code level, so that an initial judgment can be made regarding whether the app has location acquiring intentions, or not. In this stage, suspicious apps can be effectively identified and allocated a reference as to whether the apps are suspicious or not. However, since the static analysis still contains a certain misjudging rate, for further examination, a second phase of dynamic analysis is employed. As opposed to the traditional method that only checks the presence of data flow between sources to sinks within the apps’ running time, this study proposes to employ a machine learning-based idea to build a classifier model and train it to generate a more precise verdict, in the second phase. More specifically, in the first phase, the system uses the static taint method to analyze the APK file’s source code and discover whether there is a data flow from the location acquiring source APIs (Loc_Source) to data sent out APIs (Out_Sink). If it does, the system will hand it over to the second phase (dynamic analysis phase) for further examination. Once suspicious apps enter the second phase, the system exploits the prepared classifier to analyze the apps’ dynamic actions and declare its final prediction with a high accuracy rate. However, before this, there is a classifier preparation phase, which employs multiple machine learning algorithms trained with a large, labeled dataset (malware and benign apps). In order to choose the best algorithm for the classifier, multiple indicators are also established as benchmarks and compared. The whole system process diagram is shown in Figure 1, and each process will be elaborated in detail in the following subsection.

### 3.2. Static Analysis Phase

The purpose of this phase is to undertake an initial examination to target apps through the static taint analysis algorithm. For apps with positive results, the system transfers them to the dynamic procedures for further inspection. The static taint analysis algorithm aims to check whether there is a data flow from GPS acquiring APIs (Loc_Source) to the APIs of location data sent outside the terminal (Out_Sink), defined as Loc_Source and Out_Sink, respectively. Loc_Source refers to the API that acquires the user’s location information from the system, such as the latitude or longitude of the user’s location, while Out_Sink represents the API that sends the data to the outside system, such as saving logs, sending text messages, and socket connections.

Table 1 lists the Android APIs and their methods/events that belong under the definition of Loc_Source, which involves the retrieval of a user’s location data.

Meanwhile, 20 Android APIs that fit the definition of Out_Sink were also investigated, as shown in Table 2. These APIs can be divided into three categories: logging, short message service and socket connection. A malware needs to invoke some of these categories to send GPS data out of smart devices.

In order to examine the situation of the existing data flows from Loc_Resource to Out_Sink APIs, the Androguard [7] tool for static code analysis is utilized after the target APK file is decompiled. Specifically, the two modules androgexf.py and apkviewer.py are mainly used to generate the function call graphs and instruction level call graphs. With these graphs, all the connections between the two lists can be quickly found. Once the graphs show any paths from Loc_Source to Out_Sink, the classifiers conduct a further examination. Otherwise, it is assured that the target is clear since it does not involve any of the location data leakage behaviors.

### 3.3. Dynamic Analysis Phase

In the dynamic examination phase, to improve the accuracy of classification, a classifier model was first built based on machine learning technique. Specifically, it contained five procedures, which were: (1) sample dataset preparing; (2) feature selection; (3) feature extraction; (4) classifier selection; (5) establishing classifier evaluation benchmarks.

#### 3.3.1. Preparing Sample Dataset

For the purpose of the classifier’s training and testing, the sample dataset was prepared, sourced from ADM [21,22] and Androzoo [23,24]. ADM is a comprehensive dataset that contains confirmed malwares and benign apps, from which parts of malwares with location leakage risks were extracted as bad app samples. Parts of good apps were also employed from the Androzoo dataset. The two parts together formed the sample dataset.

#### 3.3.2. Features Selection

After the sample dataset was prepared, the next step was to extract their features so that the system could feed those features into the classifier for training. It was a critical step since good feature selection criteria helps to train the classifier more sufficiently. However, the criteria largely rely on researchers’ experience, and classifiers with different purposes usually have different criteria. In order to establish a comprehensive feature extraction criteria, inspired by previous studies, the following 10 features in apps were mainly considered:

(1) **The number of APIs invoked**. This reflects the comprehensiveness of an app since complex functions or modules usually need to invoke many APIs. In other words, when an app contains multiple modules and invokes many APIs, it indirectly states that the app is produced by a large company rather than a small one for a special purpose;
(2) **The number of API types**. This indicates that an app has invoked the number of distinct types of APIs, which has the same purpose as the previous criterion;
(3) **The number of geography-related API**. This depicts that an app invokes the number of APIs that involve geography operation (defined as Loc_API), such as speed, direction, longitude, and latitude. If an app calls for more Loc_APIs, it is likely that the app is providing LBSs rather than stealing GPS data;
(4) **The number of Loc_API types**. This indicates that an app has invoked the number of distinct types of geography-related APIs, which reflects the diversity of Loc_API called;
(5) **The number of Loc_Source API**. This feature evaluates the number of invoked APIs that belong to Loc_Source list in Table 1. The more Loc_Source APIs invoked, the more likely it is that the apps gathering user information are being used for normal service (benign app) or malicious purposes (insecure app);
(6) **The number of Out_Sink API**. This feature reveals the number of invoked APIs that belong to the Out_Sink list in Table 2 (e.g., network, system, and SMS). The more Out_Sink APIs calls, the higher probability that the app sends data out of the device;
(7) **The number of data flows from the Loc_Source to the Out_Sink**. Data flow or connection between Loc_Source to Out_Sink indicates that the app has a behavior of sending GPS data out of the terminal, which should draw much attention. If the number of this kind of data flow is high, it confirms that large amounts of location data have been transmitted;
(8) **The number of services**. Service is a resident thread for facilitating apps to process data in the background. Usually, malwares tend to utilize the service thread to quietly collect users’ information so that users hardly know how much location information has been obtained;
(9) **The number of keywords**. Generally, most programmers tend to use services/function/interface/method related keywords to name their apps, which inspired the authors of this study to estimate apps’ intentions by counting specific keywords. In real privacy leakage situations, the keywords are summarized into ten highly focused categories, such as navigation, social network, tourism, news, games, and shopping. Categories and their related keywords are listed in Table 3;
(10) **The size of APK files**. To some extent, the size of an APK file indicates the app’s complexity. If the size is large, it may reflect that the app has multiple modules or contains a large number of resources to fulfill many tasks. Conversely, malware usually slims itself as much as possible to encourage people to download and install it, and make it easier to be duplicated into larger premises.

#### 3.3.3. Feature Extraction

After defining the features above, Apktool.jar was utilized to decompile the APK file, which is a mature decompiling tool provided by Google. The process generates smali files, resources and configuration files, and related directories, because the smali file is the source code file under the Android platform with Dalvik as running the virtual machine. After that, Python and regular expression were used to analyze the source code file with the above-mentioned extraction features. For example, the number of APIs was calculated by thoroughly analyzing all of the smali, res/values/strings.xml, and AndroidMainfest.xml files. Nevertheless, it was still necessary to refer to the API mapping relationships between the Android API name-list and those in the smali language, since API names in smali files are not exactly the same as those on the Android API list, however, there is a mapping relationship between them. In the next step, these features are exploited to train the classifier.

#### 3.3.4. Classifier Algorithms Selection

As different machine learning algorithms have different merits, it is hard to know which is the best in this scenario. Therefore, six machine learning models were listed and various metrics (See Section 4.2) were adopted to evaluate their performance, to choose the best model to output a high-accuracy result. The six classification algorithms were: (1) **Bayesian network** [25]; (2) **decision tree** [26] (in this decision tree-based classifier, entropies ID3, C4.5 and C5.0 were used); (3) **Adaboost** [27]; (4) **random forest** [28]; (5) **support vector machine (SVM)** [29]; (6) **neural network** [30].

#### 3.3.5. Classification Result

After extracting the above features from the test app, they were fed into the classifiers for training purposes. Then the trained classifiers were able to produce a binary or float result for unknown apps by inputting its features into the classifier. There are only two outputs: positive (1) or negative (0). The positive result indicates that the malware is confirmed; while the negative result indicates that the target app is benign. As the float result output by neural networks falls into the range of [0, 1], the result of the range [0.5, 1] (positive) is considered as malware, while the rest are identified as benign.

## 4. Evaluation

In this section, the machine learning candidates described above are tested to discern which is the best model, and the methods are compared with Apparecium to show the proposal’s advancement.

### 4.1. Dataset and Classifier Implementation

In order to train and test classifiers’ performance, a dataset was prepared consisted of 12,434 malwares with location disclosure risks and 12,563 benign apps. In this dataset, with a total of 24,997 apps, the malware part was derived from the AMD dataset [22], while the benign part was derived from the Androzoo dataset [24]. Further, 6217 malwares and 6282 normal apps were randomly selected into the training dataset, while the remaining 6217 malwares and 6281 normal apps were categorized into the test dataset.

As to the implementation of the six classifiers’ algorithms, the WEKA tool (version 3.8) was employed, which provided dozens of open source machine learning algorithms. Specifically, BayesNet, J48, AdaboostM1, random forest, LibSVM and Multilayer-Perceptron algorithms in WEKA tools were utilized to implement the corresponding six classifiers of Bayesian Network, decision tree, Adaboost, RF, SVM and neural network.

### 4.2. Evaluation Benchmarks

In order to quantitatively assess the classification algorithms’ performance, six benchmarks were provided, namely: true positive rate (*TPR*), false positive rate *(FPR)*, *precision*, *accuracy*, *F-measure*, and Matthews correlation coefficient (*MCC*). The detailed definitions are as follows:

(1) $$TPR=\frac{TP}{TP+FN}$$

(2) $$FPR=\frac{FP}{FP+TN}$$

(3) $$precision=\frac{TP}{TP+FP}$$

(4) $$accuracy=\frac{TP+TN}{TP+FP+TN+FN}$$

(5) $$F-Measure=\frac{2*precision*TPR}{precision+TPR}$$

(6) $$MCC=\frac{TP*TN-FP*FN}{\sqrt{\left(TP+FP)*(TP+FN)*(TN+FP)*(TN+FN\right)}}$$

In the above definition, *TP* (true positive) and *TN* (true negative) indicates that the app has been correctly classified as a benign app or malware, respectively. *FN* (false negative) indicates that a malware is misclassified as normal. On the contrary, *FP* (false positive) indicates that a normal app is misclassified as malicious. Further, *TPR* in Formula (1) evaluates the classifier’s performance in the correct classification rate. This value is closer to 1, thus proving the better performance. On the contrary, *FPR* in Formula (2) depicts the classifier’s incorrect classification rate. When this value is closer to 0, the better performance is identified. Moreover, precision evaluates the authenticity in the classifier’s prediction results, while accuracy evaluates the ability of the classifier in correctly identifying apps. The value ranges in both precision and accuracy are between (0, 1), and when their values are closer to 1, the better performance of the classifier is demonstrated. In a different manner, F-measure is a weighted harmonic average of precision and *TPR.* The higher value of F-measure proves better classification performance. Lastly, *MCC* is similar to the correlation coefficient, and its value range is (−1, 1). Certainly, the closer the *MCC* value is to 1, the better performance of the classifier is confirmed.

### 4.3. Malware Classification Result

First of all, the 6127 malware classifications were tested with the six classifier algorithms. The test result is shown in Table 4, from which it can be seen that RF achieved the best performance, while the decision tree, SVM, and neural network output achieved nearly equal results. The Bayesian network and Adaboost performed worse than the other four algorithms.

**Figure 2:**
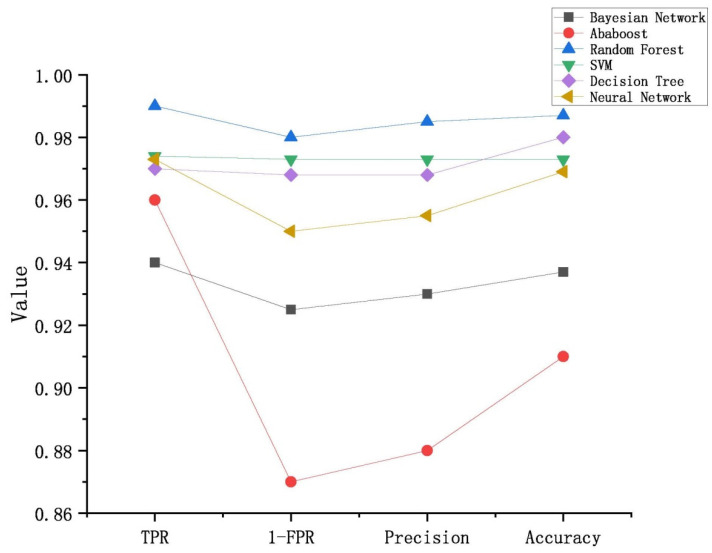
The results of malware classification for the six classifiers.

According to the results in Table 4, *TPR*, *FPR*, precision and accuracy were calculated for the six classifiers, as shown in Figure 2. It was observed that the highest accuracy was achieved by *RF* with a 99% accuracy rate on average, while the lowest accuracy was realized by the Bayesian network with a 93.9% accuracy rate.

### 4.4. Normal Apps Classification Result

The same test method was also applied to the 6281 normal apps, and the classification results are shown in Table 5. Compared with Table 4, it can be seen that the best result was achieved by *RF* with a 97.7% accuracy rate.

**Figure 3:**
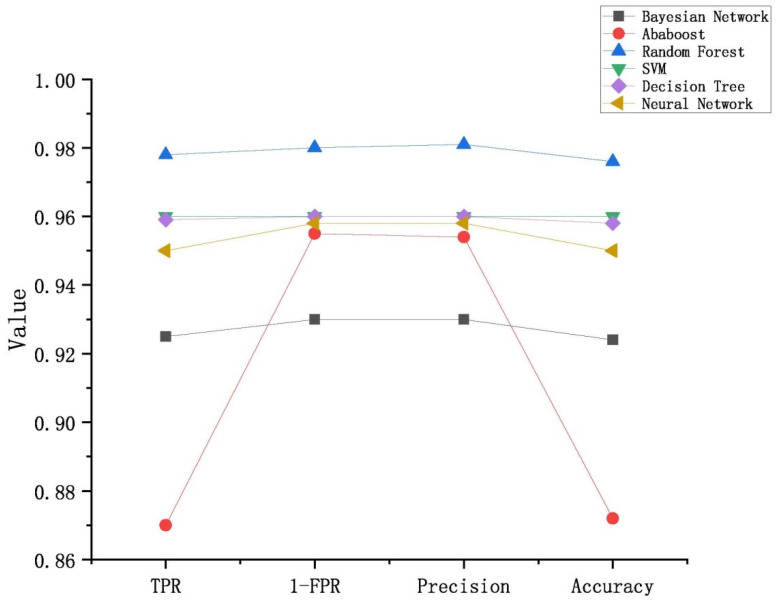
The results of normal app classification for the 6 classifiers.

Similarly, the *TPR*, *FPR*, precision and accuracy rates were calculated for the six classifiers according to the results in Table 5, which are shown in Figure 3. From this figure, it was observed that RF also achieved the best performance, while Adaboost received fluctuating results scattered in the four indicators.

**Figure 4:**
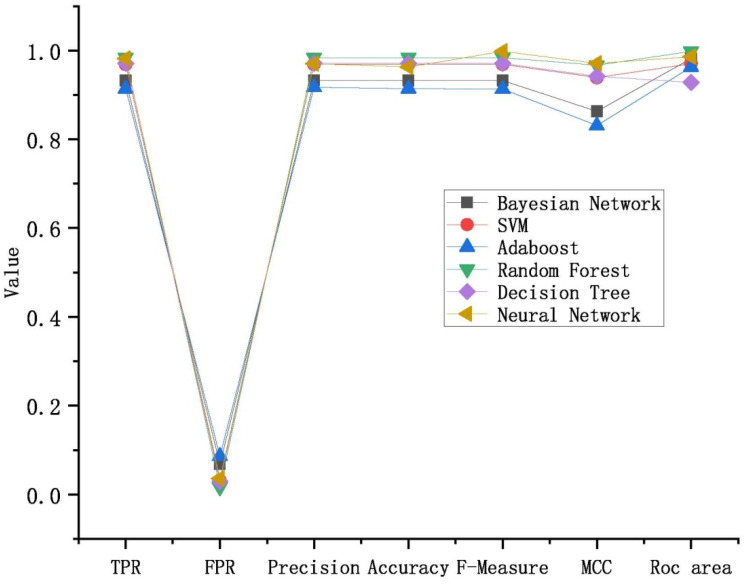
The performance of the 6 ML models with different metrics.

Consequently, based on the results in Table 4 and Table 5, a comprehensive evaluation was performed for all six classifiers to select the best, as shown in Table 6. Note that the receiver operating characteristic (*ROC*) refers to the curve that uses *TPR* as the ordinate and *FPR* as the abscissa. Thus, the *ROC* area indicates the area between the *ROC* curve and the *FPR* axis, which proves the overall classification accuracy. The closer the *ROC* area is to 1, the better classification effect of the classifier is proved. Table 6 and Figure 4 confirm that *RF* outperformed the other five classifiers with excellent results in all items, and achieved a 98.4% accuracy rate on average.

### 4.5. Comparison with Classic Schemes

**Figure 5:**
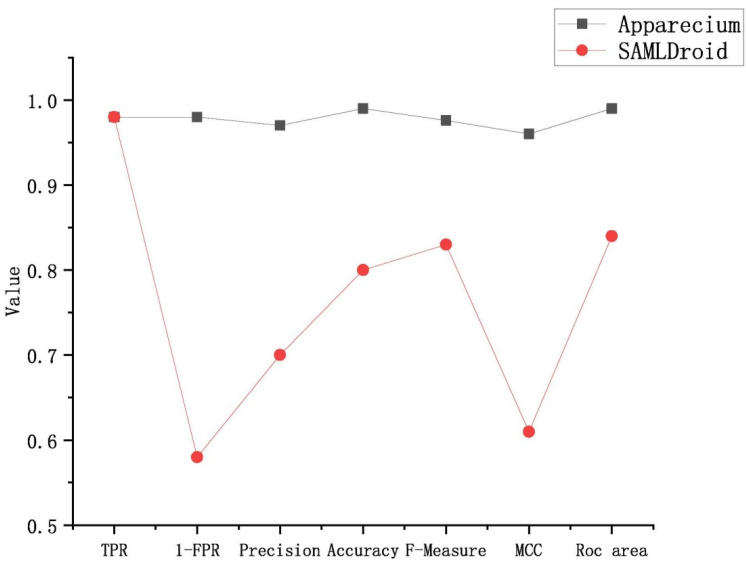
The comparison between this proposal and Apparecium.

In order to prove the advanced performance of this proposal, Apparecium was reimplemented and the proposal methods compared with six metrics, as shown in Figure 5. The results confirmed that the proposal achieved the highest overall classification effects, up to 98.4%, on average. Its performance was about 20% higher than Apparecium in the items of *FPR*, precision, accuracy, F-measure, *MCC* and *ROC* area.

## 5. Conclusions

Nowadays, mobile apps with location services are thriving and the problem of location privacy leaks emerges significantly, posing a great threat to mobile social network users. However, traditional methods including either static code analysis or dynamic behavior analysis have failed to identify malwares with a high accuracy. In order to improve the identification accuracy for apps with location privacy leakage risks, this paper proposed a combined static analysis and machine learning method for the Android platform, SAMLDroid. Briefly, SAMLDroid first uses static code analysis to scrutinize target apps’ source code and undertake a fast and preliminary examination to check for the existence of a data path from location data retrieving APIs to data sending-out APIs. If suspicious, SAMLDroid then uses machine-learning to conduct a behavior level examination to output a precise result. Meanwhile, the selection of the best classifier was undertaken by a thorough evaluation using six well defined indicators, which confirmed that RF had the best performance. Eventually, it was proven that the SAMLDroid method possessed higher accuracy in apps classification, namely, up to 98.4%, surpassing the accuracy of Apparecium by nearly 20%. However, not every data flow was monitored regarding GPS data or analysis of the action pattern in the app running state, because more accuracy incurs larger cost. Future research plans are to integrate more features to improve accuracy.

## Figures and Tables

**Table 1:** The location-acquired APIs and their methods (Loc_Source API list).

ID	APIs	Methods or Events
1	Landroid/location/Location	getLatitude()
2	Landroid/location/Location	getLongitude()
3	Landroid/location/LocationListener	onLocationChanged()
4	Landroid/location/LocationManager	getLastKnownLocation()
5	Landroid/telephony/gsm/GsmCellLocation	getLac()
6	Ljava/util/Calendar	getTimeZone()
7	Ljava/util/Locale	getCountry()
8	Landroid/telephony/TelephonyManager	getCellLocation()

**Table 2:** The location data-sending-related APIs (Out_Sink API list).

ID	APIs	Methods or Events
1	Landroid/os/Handler	sendMessage()
2	Landroid/telephony/SmsManager	sendDataMessage()
3	Landroid/telephony/SmsManager	sendMultipartTextMessage()
4	Landroid/telephony/SmsManager	sendTextMessage()
5	Landroid/util/Log	d()
6	Landroid/util/Log	e()
7	Landroid/util/Log	i()
8	Landroid/util/Log	v()
9	Landroid/util/Log	w()
10	Landroid/util/Log	wtf()
11	Ljava/io/FileOutputStream	write()
12	Ljava/io/OutputStream	write()
13	Ljava/io/Writer	write()
14	Ljava/net/Socket	connect()
15	Ljava/net/URLConnection	setRequestProperty()
16	Ljava/net/URL	init()
17	Ljava/net/URL	set()
18	Lorg/apache/http/client/HttpClient	execute()
19	Lorg/apache/http/impl/client/DefaultHttpClient	execute()
20	Lorg/apache/http/message/BasicNameValuePair	init()

**Table 3:** The keywords list.

ID	APIs	Keywords in the Source Code
1	Navigation	Navigation, map, GPS
2	Social	Social, communication, social, make friends, marriage, community, chat
3	System tools	Root, landscaping, root, file management, optimization, energy saving, system, wifi, typewriting, download, cloud disk, system, tool, mailbox, email, browser
4	Multi-Media	Media, video, camera, photo, picture, live, TV, site, web, film, television, wallpaper, retouching, bell, radio, e-books, KTV, song, books
5	Education	Education, learning, curriculum, letters, English, backwords, examination, office
6	Finance	Economic, bank, lottery, driving test, payment, stock, bank, finance, investment, insurance
7	Tourism	Travel, traffic, aircraft, train, bus, translation, ticket
8	News	News, bulletin, wall bulletin, stop-press news, newsflash, information
9	Game	Entertainment, music, game, Mobile Games, cartoons, sports, gaming
10	Shopping	Consumption, takeout, takeaway, shopping, group buying, car rental, electricity

**Table 4:** The identification results of 6127 malware with 6 classifiers.

ML Models	Malware Number	Correct Classification	Incorrect Classification	Correct Classification Rate
Bayesian network	6217	5840	377	93.94%
Decision tree	6217	6061	156	97.49%
Adaboost	6217	5957	260	95.82%
Random forest	6217	6157	60	99.03%
SVM	6217	6050	167	97.31%
Neural network	6217	6019	138	96.82%

**Table 5:** The identification results of 6127 normal apps with 6 classifiers.

ML Models	Normal App Number	Correct Classification	Incorrect Classification	Correct Classification Rate
Bayesian network	6281	5802	497	92.37%
Decision tree	6281	6053	228	96.37%
Adaboost	6281	5461	820	86.94%
Random forest	6281	6137	144	97.7%
SVM	6281	6085	196	96.88%
Neural network	6281	5968	313	95.02%

**Table 6:** The performance of the six ML models with different metrics.

ML Models	TPR	FPR	Precision	Accuracy	F-Measure	MCC	Roc Area
Bayesian network	0.932	0.068	0.932	0.932	0.932	0.863	0.982
Decision tree	0.969	0.031	0.969	0.969	0.969	0.939	0.970
Adaboost	0.914	0.086	0.917	0.914	0.913	0.831	0.963
Random forest	0.984	0.016	0.984	0.984	0.984	0.967	0.998
SVM	0.971	0.029	0.971	0.971	0.971	0.942	0.971
Neural network	0.964	0.036	0.964	0.964	0.964	0.928	0.986

## Data Availability

Not applicable.
